# Many Faces of Renin-angiotensin System - Focus on Eye

**DOI:** 10.2174/1874364101711010122

**Published:** 2017-06-19

**Authors:** Mervi Holappa, Heikki Vapaatalo, Anu Vaajanen

**Affiliations:** 1 BioMediTech, University of Tampere, Tampere, Finland; 2 Medical Faculty, Department of Pharmacology, University of Helsinki, 00014 Helsinki, Finland; 3 Department of Ophthalmology, Tampere University Hospital, Tampere, Finland; 4 SILK, Department of Ophthalmology, School of Medicine, University of Tampere, Tampere, Finland

**Keywords:** ACE1, ACE2, Angiotensin II, Angiotensin (1-7), Eye, Glaucoma, Intraocular pressure, Mas receptor, Renin-angiotensin system

## Abstract

The renin-angiotensin system (RAS), that is known for its role in the regulation of blood pressure as well as in fluid and electrolyte homeostasis, comprises dozens of angiotensin peptides and peptidases and at least six receptors. Six central components constitute the two main axes of the RAS cascade. Angiotensin (1-7), an angiotensin converting enzyme 2 and Mas receptor axis (ACE2-Ang(1-7)-MasR) counterbalances the harmful effects of the angiotensin II, angiotensin converting enzyme 1 and angiotensin II type 1 receptor axis (ACE1-AngII-AT1R) Whereas systemic RAS is an important factor in blood pressure regulation, tissue-specific regulatory system, responsible for long term regional changes, that has been found in various organs. In other words, RAS is not only endocrine but also complicated autocrine system. The human eye has its own intraocular RAS that is present *e.g.* in the structures involved in aqueous humor dynamics. Local RAS may thus be a target in the development of new anti-glaucomatous drugs. In this review, we first describe the systemic RAS cascade and then the local ocular RAS especially in the anterior part of the eye.

## INTRODUCTION

Glaucoma, known for its destructive effects on the optic nerve axons and retinal ganglion cells, is one of the leading causes of blindness worldwide. In the next three years, approximately 80 million people around the world are predicted to be diagnosed with glaucoma [1]. This number is likely to be even higher due to the fact that glaucoma can be asymptomatic for a long time which makes it difficult to detect until it is severe [2, 3]. Glaucoma is a multifactorial optic neuropathy that can be roughly divided into two categories: open-angle glaucoma (POAG) and angle-closure glaucoma, both of which can cause visual field defects and irreversible vision loss [4-10].

 While many genetic and biological risk factors have been identified, such as age, race and diabetes, the most fundamental reason why a patient is diagnosed with glaucoma is increased intraocular pressure (IOP) [6, 10-13]. The exact underlying mechanism for glaucoma development is still poorly known which makes it difficult to develop effective therapies for this destructive disease [14, 15]. As of today, the only treatment demonstrated to be effective to reduce disease progress is to lower IOP [16, 17]. That is to say, decrease of IOP by 30% reduces disease progress [18, 19]. In addition to the classical RAS that regulates blood pressure (BP), fluid volume as well as electrolyte balance and is also involved in inflammation, local tissue-specific RA-systems have been described in numerous organs including the human eye [20-24]. While the pathological mechanisms at molecular level are not yet well understood, local ocular RAS seems to have a role in ocular pathology and it has been associated to glaucoma and other eye disorders such as diabetic retinopathy (DR), age-related macular degeneration (AMD) and retinopathy of prematurity (ROP) [22, 25]. In this review, we describe the local ocular RAS cascade and concentrate on its potential role in the regulation of IOP while keeping the focus on the anterior part of the eye. A comprehensive survey of literature on PubMed was performed to collect the literature and eventually 258 articles were chosen based on their relevance. The following keywords were used and combined to narrow down the literature: IOP (41729), RAS (29132), tissue RAS (6080), angiotensin (116858), angiotensin I (8316), angiotensin II (59230), angiotensin (1-7) (1278), angiotensin (1-9) (73), angiotensin (3-4) (10) angiotensin converting enzyme 1 (238) and -2 (1453), Mas receptor (445), angiotensin receptor (17975), eye disease (5611), glaucoma (59565), diabetic retinopathy (DR) (26740), retinopathy of prematurity (ROP) (6638) and age-related macular degeneration (AMD) (13303).

## RENIN-ANGIOTENSIN SYSTEM

### Circulating Ras

The renin-angiotensin system is one of the oldest and most studied peptide cascades known today. The research concentrating on RAS started, in 1898, in Finland when Robert Tigerstedt and Per Bergman identified a renal BP elevating substance: renin [20, 26, 27]. Over 40 years later, more components of the RAS cascade were identified, one being the angiotensin peptide, first named as angiotonin, that Braun-Menéndez and Page reported to be formed from angiotensinogen by renin, an enzyme originated in the kidney [28-30]. 1970 was an important year in the RAS research as its role in BP and fluid balance regulator was understood which led to the development of the first antihypertensive medications such as captopril that blocks the formation of Ang II by inhibiting ACE1 [20, 28]. Later more antihypertensive drugs were designed and developed, one of which was Ang II type 1 receptor blocker (ARB) that blocks the vasoconstrictive effects of Ang II through its main receptor [31].

**Fig. (1):**
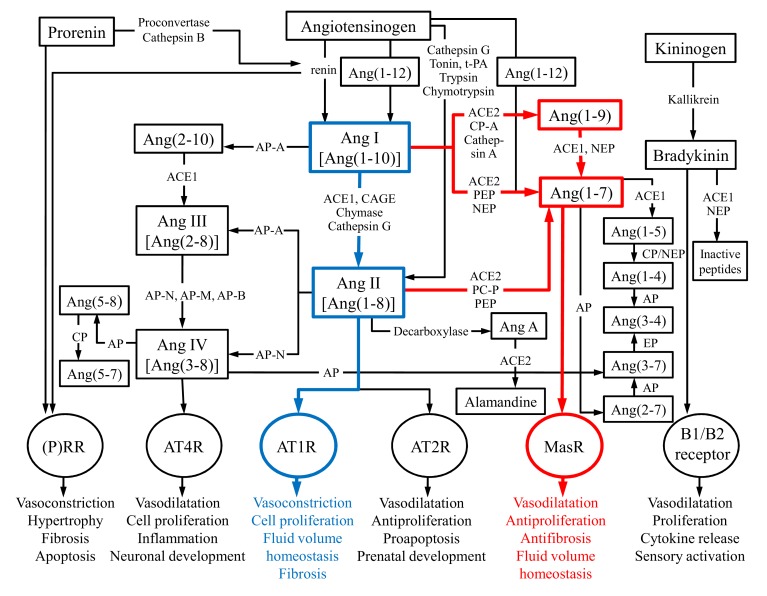
The renin-angiotensin system. The two main axes of RAS are highlighted with colors. ACE2-Ang(1-7)-MasR axis (red lines) counterbalances the harmful effects of the ACE1-Ang II-AT1R axis (blue lines). ACE1: Angiotensin-converting enzyme 1; ACE2: Angiotensin-converting enzyme related carboxypeptidase; Ang I, II, III, IV: Angiotensin I, II, III, IV; Ang A: Angiotensin A; AT1R, -2R, -4R: Angiotensin II type 1, -2, -4 receptor; AP: Aminopeptidase (-A, -N, -M, -B); B1/B2: Bradykinin receptors; CAGE: Chymostatin-sensitive Ang II generating enzyme; CP: Carboxypeptidase; EP: Endopeptidase; Mas receptor: Ang(1-7) receptor type; NEP: Neprilysin; PEP: Prolyl-endopeptidase; PCP: Prolyl-carboxypeptidase; tPA: Tissue-type plasminogen activator. In angiotensin peptides the numbers in parenthesis refers to the numbers of amino acid residues. The figure is updated from Vaajanen *et al.* [41].

Multiple peptides, enzymes and receptors have been identified since the first clue of the existence of RAS. One of the important and fairly recent findings was the identification of a new receptor type: MasR whose activities, such as vasodilatation, antifibrosis and antiproliferation, are opposite to those of angiotensin II type 1 receptor (AT1R). This discovery led to the division of RAS into two central axes: ACE1-Ang II-AT1R and ACE2-Ang(1-7)-MasR giving antihypertensive drug development new angles to focus on [32-34] as chronic imbalances of these main axes can potentially lead to pathological events of *e.g.* renal, cardiovascular and central nervous system [35]. In time, RAS has truly evolved from simple linear pathway, having only one known substrate, two proteases, two peptides and one receptor, into a complex cascade consisting of multiple mediators, functionally versatile enzymes and various receptors that are activated by several angiotensin peptides [36-40]. Figure 1 shows the full extent of the RAS cascade known today.

The classical RAS cascade starts with highly specific aspartyl protease renin that cleaves the peptide bond between Leu10 and Val11 at the amino terminus of angiotensinogen (AGT), a 225 amino acids long α-glycoprotein, to form angiotensin I (Ang I) [28, 42, 43]. AGT is mainly synthesized and released from liver but other organs such as heart, kidney and adipose tissue can also produce it [44]. Inflammation, insulin and estrogens among other things can stimulate the synthesis of AGT [42]. Renin is mainly synthesized in kidney as an inactive prorenin that is activated by either cathepsin B or proconvertase and secreted from the juxtaglomerular apparatus [28, 42, 43] in response to either decreased arterial BP, decreased sodium levels or increased activity of sympathetic nervous system [45-47]. Both renin and prorenin can bind to (pro)renin receptor ((P)RR) and thus mediate vasoconstrictive effects [42, 48].

Ang I, a weak prohormone and vasoconstrictor can be further cleaved to form Ang II. Different enzymes can form Ang II from Ang I: *e.g.* ACE1, tonin [49], trypsin [50], kallikrein [51], cathepsin G [52] and chymase [53-55]. ACE1 is present in many tissues as well as in biological fluids *e.g.* in plasma [56-58]. In order to work, ACE1 needs Zn^2+^ in complex with activated water molecule in its active site [59] and chloride to improve substrate binding [60]. This main enzyme acting on Ang I, removes two amino acids (His-Leu) from the carboxyl terminus of Ang I to form one of the central peptides of RAS cascade: Ang II. In addition to being an important enzyme in RAS, ACE1 also acts in kallikrain-kinin system [42, 45]. Other enzymes mentioned above are regarded as alternative pathways for Ang II generation [61-63] which are important in physiological and pathophysiological conditions [64, 65]. However, these pathways are not discussed further in this review.

In 1940, Ang II an octapeptide also known as Ang (1-8) was first isolated and characterized as a potent vasoconstrictor that raises BP and regulates *e.g.* electrolyte balance, vascular tone and thirst [29, 30, 43]. It stimulates the release of aldosterone and vasopressin and exerts its harmful actions, such as vasoconstriction, fibrosis and inflammation *via* G-protein coupled AT1R [42, 43, 66-68]. Furthermore, Ang II can also activate angiotensin II type 2 receptors (AT2R) whose activities are thought to oppose those of AT1R [31, 42, 43]. AT2Rs are regarded as protective receptors as they may elicit vasodilatory, antihypertensive, proapoptotic as well as antiproliferative effects [53, 69] and as they can bind directly to AT1R thus inhibiting signaling through it [31]. ACE inhibitors are used as antihypertensive medications as they oppose the harmful effects of Ang II *via* AT1R by blocking the conversation of Ang I to Ang II by ACE1 thus elevating the levels of Ang(1-7) [42, 43].

Angiotensin II can then be further cleaved by ACE1 or aminopeptidase A to generate angiotensin III (Ang III) or by aminopeptidase N to form angiotensin IV (Ang IV) [20, 28, 42, 43]. Ang IV can also be generated from Ang III by aminopeptidases N, M and B [28, 43]. Ang III exerts its vasoconstrictive actions *via* the same receptors as Ang II (higher affinity to AT2R) whereas Ang IV prefers angiotensin II type 4 receptors (AT4R) which are related to cognitive functions and proliferative effects and which are found *e.g.* in brain, lung and kidney [20, 70, 71]. Ang IV can also elicit its biological effects, including renal vasodilatation, hypertrophy and regulation of cell growth, through activation of AT1R [72].

Cleavage of one amino acid residue (Phe) from carboxyl terminus of Ang I by ACE2 [73], carboxypeptidase A or cathepsin A [74, 75] generate angiotensin (1-9) (Ang(1-9)) whose main biological functions are to release arachidonic acid, promote nitric oxide formation and increase bradykinin activity [74]. Ang(1-9) may also reduce BP, decrease hypertension [76] and possibly play a role in inhibition of platelet function [77]. The formation of Ang(1-9) is thought to be dependent on ACE2 activity [73, 78] and it has been suggested that Ang(1-9) could mediate its actions by activating the AT2 receptors [76, 79]. ACE2 (42% sequence identity to ACE1) was first cloned in 2000 and has since been identified in multiple organs such as kidney, heart, brain, liver and lung [42, 73, 80-82]. ACE2 has been shown to convert Ang I to Ang(1-9) [73] and most importantly to form Ang(1-7) from Ang II [42, 80, 83-85]. This enzyme has zinc metallopeptidase consensus sequence (HEXXH) in its active site and like its homologue ACE2 activity is also regulated by the presence of chloride ions [42]. ACE2 cleaves only one amino acid residue from Ang II thus enhancing Ang(1-7) formation and leaving less substrate to ACE1 to act on [42, 82]. Furthermore, ACE inhibitors used as antihypertensive medications do not block ACE2 activity making this enzyme together with Ang(1-7) and MasR the focus of the research regarding RAS and cardiovascular drug development [80, 86].

Ang(1-7) that was first thought to be devoid of biological function can be generated from Ang II by ACE2, prolyl-endopeptidase and prolyl-carboxypeptidase [20, 43, 80, 87] or from Ang(1-9) by ACE1 and NEP [83]. It can also be metabolized directly from Ang I or from prohormone Ang(1-12) bypassing the biosynthesis of Ang II [43, 87]. Moreover, Ang(1-7) can be further metabolized into smaller peptides such as angiotensin (1-5) or angiotensin (2-7) [28].

Ang(1-7) is known to have functions opposite to those of Ang II [43]. Although, Ang(1-7) may interact with AT1R and AT2R [76, 79, 84, 86], this peptide elicits its vasodilating and antiproliferatory effects through activation of its main receptor MasR [20, 42, 79, 84]. MasR is a G-protein coupled receptor [88] that was first described as a proto-oncogene [89]. This receptor has been found *e.g.* in eye, central nervous system, kidney, heart and brain [88, 90-93] and is known to act antagonistically to AT1R [53]. Due to its antiarrhythmogenic, antithrombogenic, growth-inhibitory and vasoconstrictive inhibitory properties, Ang(1-7) is seen as a protective peptide that acts as a counterregulator within RAS [38-40]. Interestingly, the association between Ang(1-7) and pathology of multiple diseases such as hypertension [84, 86, 87, 94-98] and diabetic nephropathy [46, 99, 100] makes this peptide a potential target for drug development.

Interestingly, in addition to ACE2-Ang(1-7)-MasR axis, a newly described member of RAS, angiotensin(3-4) (Ang(3-4)) counteracts the traditional ACE1-Ang II-AT1R pathway both systemically and locally[101]. Ang(3-4) has antihypertensive effects that are mediated *via* AT2 receptors. This dipeptide is antiproliferative and vasodilating and it inhibits ACE1. Furthermore, it is effective when administered orally, as it permeates intestinal cells relatively easily, lowers Ang II and aldosterone levels in plasma and inhibits ACE1. Ang(3-4) levels are higher in healthy individuals than in hypertensive patients [101]. All in all, it remains open what are the therapeutic possibilities of Ang(3-4) and its potential functions in local RA-systems beyond renal tissue such as in eye.

In addition to previously mentioned angiotensin peptides and enzymes, there are several different peptidases, proteases and small peptides that are part of the RAS cascade but are not discussed in this review. However, all of these enzymes and smaller angiotensin peptides are shown in Fig. (**1**). In addition, the kallikrein-kinin system that is also shown in Figure 1 and that interacts with RAS is not discussed in detail in this review.

## TISSUE RAS

Even though circulatory RAS has many important roles in human body, various organs have their own tissue specific RA-systems that elicit long term changes and local effects such as growth, proliferation and protein synthesis at organ level [20, 31, 64]. Ganten *et al.* 1971 were the first researchers to show that RAS is also an organ specific system that has important regulatory roles at tissue level [102]. For example, heart, brain, intestine and even the human eye have their own local RA-systems [6, 31, 43].

Later, local RA-systems were divided into two groups based on the origin of Ang II: extrinsic RAS gets its Ang II from the circulation whereas intrinsic RAS synthesises its own Ang II locally [103]. Although some of the local RA-systems depend on interactions with circulatory RAS to fully operate, in many organs tissue RA-systems prefer to function independently [43]. Indeed local tissue-specific RA-systems can independently produce different components of RAS thus proving that RAS is more than just an endocrine circulatory system and that it regulates more functions than suggested earlier [6, 31, 43, 64].

Local ocular RAS has been found partly in the eye. So far all of the central components of RAS including the components of the two main axes: ACE1-Ang II-AT1R and ACE2-Ang(1-7)-MasR have been identified in different eye structures in various species [6, 9]. In the human eye, elements of the two main axes have been identified in retinal as well as in non-retinal ocular structures [6, 93, 104]. Tables (**1** and **2**) summarize the localization of RAS peptides, enzymes and receptors in non-retinal ocular structures of the human eye.

Even though RAS is present in the human eye, its function and significance in ocular pathophysiology is still unknown. Whether angiotensin peptides found in the human eye originate partially from the blood compartment or are synthesized locally has been the topic of the debate [117]. Circulating angiotensins *e.g.* Ang I and Ang II cannot pass the blood-brain barrier and cannot reach the vitreous fluid when blood-retina barrier in the eye is intact [24, 117, 128, 129]. Moreover, in porcine eyes Ang I and Ang II levels are shown to be 5 to 100-fold higher than those found from diffusion of blood [117] and in comparison to plasma ACE1 activity are higher in ocular structures of pig and rabbit eyes [130, 131]. However, intraocular RAS may play a role in the regulation of IOP through its effects on AH dynamics [9, 31]. Ang II has been suggested to increase AH secretion *via* AT1R [122]. Animal studies as well as studies on different patient groups indicate that systemic antihypertensive RAS-inhibiting medications such as ACE inhibitors [103, 132, 133], ARBs [134-136] and renin inhibitors [137], reduce IOP. Moreover, intraocular RAS and its actions have been linked to various eye diseases [6, 138].

## EYE DISEASES AND LOCAL RAS

### Glaucoma

Glaucoma is a multifactorial long-term neurodegenerative disorder which can be characterized by the non-apoptotic and apoptotic death of retinal ganglion cells and the loss of retinal nerve fibers all of which lead to loss of visual field [6, 7, 9, 10]. The death of retinal ganglion cells leads to increased IOP which is the most important risk factor for the development of glaucoma and the only risk factor amenable to treatment [15, 138, 139]. Increased IOP can lead to ischemia, mechanical impairment, oxidative stress and optic nerve inflammation [140]. However, not every patient with high IOP develops glaucoma nor do all the glaucoma patients have increased IOP [15]. Several other risk factors such as age, family history, diabetes, vascular dysfunction and systemic hypertension have also been linked to glaucoma [6, 9-11, 139, 141-144]. In addition to genetic and environmental factors, epigenetics also affect the signaling pathways that are held responsible for glaucoma progression [145, 146].

IOP is maintained by a homeostatic balance between formation and outflow of AH. In the healthy human eye, the flow of AH against resistance generates an IOP of about 15 mmHg (range 9-21 mmHg) [147-149]. Diurnal variation of IOP is about 5 mmHg in healthy subjects. Higher IOP values are normally measured in the morning. Transient postural IOP variations can be even twofold *e.g.* in yoga practioners during sirsasana, headstand posture [150]. In addition, physical exercise can influence ocular pressure [151, 152]. Typically aerobic exercise reduces IOP due to the better ocular circulation [153] whereas anaerobic exercise elevates IOP due to the transient obstructed ocular circulation [154]. The pressure is needed to maintain the optical and refractive properties of the eye [148, 149, 155]. One of the other fundamental functions of IOP is to maintain the right shape of the eye [148, 155]. The normal AH formation rate (2.5-2.8µL/min) is lower while sleeping (1.5µL/min) and can be reduced with ageing and in some systemic diseases such as in diabetes [9, 139, 156].

AH is a mixture of different electrolytes, growth factors, proteins, amino acids, cytokines, inorganic and organic solutes [157-161]. Circulating AH not only sustains and nourishes non-vascularized eye structures *e.g.* cornea and lens but it also removes excretory products, transports neurotransmitters and enables mediators and inflammatory cells to circulate in the eye [9, 156]. Ciliary body epithelial is the site of AH production [147]. Although two passive processes: diffusion and ultrafiltration, that require no cellular activity nor energy [148], also participate on AH production [162], active secretion accounts for 80-90% of the total AH formation [155, 156, 163]. Active and selective trans-cellular transport of ions and molecules across the epithelium against concentration gradient requires energy that is generated by hydrolysis of adenosine triphosphate (ATP) by Na^+^/K^+^ ATPase. Na^+^ and K^+^ activate ATP hydrolysis whereas different molecules such as dinitrophenol and vanadate inhibit Na^+^/K^+^ ATPase [156, 164, 165]. Moreover, active transport of Na^+^ into the posterior chamber causes water flow from the stromal pool into the posterior chamber and two aquaporins (AQP1 and AQP4) have been shown to contribute to AH formation [166, 167]. Other ions and molecules that are actively transported across the epithelium include Cl^-^, HCO3^-^, ascorbic acid and certain amino acids. However, active transport of these components occur to a lesser extent [156, 166, 168-170]. After the production and secretion into the posterior chamber, AH flows between the lens and the iris into the anterior chamber from where it will be disposed [157, 171, 172].

From the anterior chamber, AH flows through the trabecular meshwork and the canal of Schlemm into the venous blood system [147]. AH can exit the eye through the trabecular, the uveoscleral or the uveolymphatic pathways [139] from which the first is pressure-dependent main route of drainage accounting for 90% of all AH outflow [9, 139, 173]. Aqueous humor flows through the porous trabecular meshwork passively when appropriate IOP level is reached and AH gets filtered in the process [174]. The actin cytoskeleton and the adhesions of trabecular meshwork cells affect the fluid outflow through the trabecular meshwork [175]. The first route of drainage can also be described as the conventional pathway [176] which rate limiting step is the flow through the inner wall of Schelmm's canal [58]. From Schelmm's canal dozens of collector channels connect with aqueous veins through which AH flows into the circulatory system [177, 178].

In addition to trabecular pathway some AH exit the eye through the uveosscleral route [179]. In comparison to the trabecular pathway the uveoscleral pathway is relatively independent of IOP and undergoes age-dependent changes [173, 179]. The uveoscleral route of drainage can also be described as the unconventional pathway in which AH drains through the ciliary muscle and exits through the supraciliary space across the anterior/posterior sclera into the choroidal vessels and returns to systemic circulation [180, 181]. The rate limiting step in uveoscleral pathway is the flow through the ciliary muscle [179].

The third route of drainage that is thought to work as a backup system is located in channels of the ciliary body stroma and intestinal space between ciliary muscle bundles [182]. Other minor outflow pathways *via* iris vessels, corneal endothelium and anterior vitreous body have also been described [183]. As IOP is the net sum of aqueous humor formation and outflow, anti-glaucomatous treatments aim to lower IOP by either decreasing the rate of AH formation or by increasing AH drainage [184]. Lowering IOP by laser therapy, ocular hypotensive medications or surgical procedures is currently the only therapeutic tool to treat this devastating disease [6, 9, 10, 28].

Some studies have suggested that drugs affecting RAS by blocking its action *e.g.* ACE inhibitors [132, 133, 185] and ARBs [134, 135] might be potential anti-glaucomatous drugs in the future. Animal studies also support these findings [103, 133, 135-137]. ACE inhibitors can affect IOP levels through their actions on AH dynamics. These inhibitors can decrease Ang II levels in AH, thus affecting the uveoscleral outflow [118, 186, 187], and slow down AH formation by lowering blood flow in the ciliary body [188] which is the primary site of AH production. ACE inhibitors also act through kallikrein-kinin system. By blocking the breakdown of bradykinin these inhibitors support prostaglandin synthesis which in turn lowers IOP by elevating uveoscleral outflow [189-191]. By preventing bradykinin breakdown ACE inhibitors also cause vasodilatation as their actions lead to increased nitric oxide formation by endothelial cells. Both prostaglandins and nitric oxide being vasodilatory also inhibit the synthesis of vasoconstrictive peptide endothelin-1 [192-195]. In addition, ARBs have been suggested to elevate uveoscleral outflow and slow down cell death of retinal ganglion cells [195, 196].

RAS components have also been identified in central structures of the eye responsible for AH formation and RAS activity has been reported in cultured human non-pigmented ciliary epithelial cells [6, 120, 122]. Interestingly, Ang(1-7) that acts *via* MasR and ACE2 activating diminazene aceturate (DIZE) have been reported to have positive effects on glaucoma by decreasing IOP [41, 197, 198]. Indeed, as Ang(1-7) actions are opposite to those of Ang II, this heptapeptide is thought to have beneficial effects on the human body including the eye. Moreover, ACE2 activating compounds are considered as novel pharmacotherapeutic agents [7, 41, 199-201].

Whereas Ang(1-7) and ACE2 activation are thought to have positive effects on IOP, Ang II has suggested to have negative effects on the human eye even though some of the results are controversy. Ang II augments cell proliferation in trabecular meshwork and increases collagen synthesis in vivo [187]. Intracamerally administered Ang II also lowers uveoscleral outflow [41] whereas in cats intravenously administered Ang II decreases IOP [202]. Ang II can also act as a secretagogue peptide in non-pigmented ciliary cells [122] as it promotes potassium ion channel activity through Ca^2+^ signaling [203] and causes higher cytoplasmic sodium concentration by activating Na^+^/H^+^ exchange [163]. Imbalances in sodium handling in ciliary and renal tubular epithelium might explain the association between glaucoma and hypertension [204]. On the other hand, defects in autoregulation of the posterior ciliary circulation [205] and microvascular damage affecting the optic nerve blood supply [206] both caused by hypertension could also explain the coexistence of hypertension and glaucoma. Furthermore, hypotensive periods caused by antihypertensive medications can also lead to injured optic nerve fibers [207].

## Diabetic Retinopathy

Diabetic retinopathy (DR) is one of the most leading causes of blindness worldwide and one of the most common microvascular complications of diabetes [208-210]. As diabetes becomes more and more common among adults, the incidences of DR are predicted to rise dramatically. Nearly all patients with type 1 diabetes and almost 60% of patients suffering from type 2 diabetes show some signs of DR later in diabetes [211]. Many factors such as high BP, hyperglycemia, hyperlipidemia, age and oxidative stress play a role in the development of DR [212, 213]. The early state of the disease is described as non-proliferative DR (NPDR) in which microaneurysms are formed due to the weakened retinal blood vessels and blood-retinal barrier breaks down. After the formation of microaneurysms, fluid can leak into retina causing swelling of the macula. Proliferative DR (PDR) is used of more serious and advanced form of DR. In addition to the symptoms seen in NPDR, PDR also manifests blood vessel growth on the surface of the retina and into the vitreous [209, 214, 215].

Studies have shown that RAS, especially Ang II mediated actions through AT1R, is involved in the progression of DR [31, 52, 94, 216-218]. Over-expression of Ang II and prorenin as well as vascular endothelial growth factor (VEGF) exists in vitreous humor of individuals suffering from DR [24, 219-221]. Ang II promotes angiogenesis and possibly increases the risk of neovascularization and hyperpermeability by increasing the retinal blood vessel permeability [219, 220, 222-225]. Furthermore, (P)RR has been linked to the development of DR through its direct effects on expression of angiogenic molecules in retinal cells [226-229]. RAS involvement in angiogenesis has promoted research concentrating on the potential therapeutic role of RAS inhibitors in DR [230]. Blocking the RAS cascade can actually slow down the progression of the disease [231-233] especially in normotensive patients [234]. In patients with type 1 and type 2 diabetes, a link between decreased blood pressure and reduced DR progression has been described [235, 236]. RAS inhibitors can reduce the risk of DR and possibly affect the progression of the disease in diabetic patients [229]. In addition, ACE2 activation reduces retinal ganglion cell death in hyperglycemic rats [198]. Afterall, more basic research and randomized clinical trials are needed in the future to resolve the exact mechanism of action of RAS compounds.

## Age-related Macular Degeneration

Age-related macular degeneration (AMD) is a major cause of irreversible vision loss among aged population. This devastating disease is the reason why 8.7% of people worldwide suffer from blindness [237]. Whereas dry AMD is characterized with the significant loss of photoreceptors leading to the loss of central vision, wet form of AMD causes choroidal neovascularization in which pathologically grown blood vessels penetrate Bruch's membrane and populate retina. 90% of AMD cases are diagnosed as dry form of AMD. Interestingly, in addition to many risk factors such as old age, smoking, systemic hypertension as well as environmental and genetic factors imbalances in RAS cascade have also been connected to the development of AMD [6, 22, 208, 238-241].

Circulating RAS is known for its role as the regulator of BP and defects in RAS cascade can lead to systemic hypertension which in turn raises the risk of developing AMD. Furthermore, Ang II modulates retinal pigment epithelium which suggests that impairment in RAS regulation might affect the function of retinal pigment epithelium as well as the viability of photoreceptors. Ang II affects retinal angiogenesis which in turn suggests that this vasoconstrictive octapeptide might also be involved in choroidal neovascularisation [6, 238]. The activation of two types of RAS receptors: AT1R and (P)RR have also been linked to the development of AMD [242, 243]. Animal studies have shown that losartan an AT1R antagonist [244], ARBs [245], ACE inhibitors [239] and (P)RR inhibitors [243] have positive effects on AMD as these compounds can reduce choroidal neovascularization by suppressing inflammatory agents. As of today, no effective therapies are available for treatment of dry AMD [246]. However, understanding the role of RAS in the development of AMD might help us manage and slow down the progression of this disease [138, 247].

## Retinopathy of Prematurity

Whereas AMD affects elderly people, retinopathy of prematurity (ROP) is an eye disease that only affects premature newborns (born <32 weeks) and can lead to permanent vision loss [25, 238]. The main risk factors identified for development of ROP are lower gestational age and low birth weight (<1.5 kg). In industrialized countries, roughly two-thirds of newborns with birth weight less than 1.25 kg manifest some signs of ROP. All the risk factors identified for this disease correlate with retinal immaturity at birth [25, 248]. ROP can be characterized as neovascular disease in which retinal neovascularisation leads to several complications such as macula dragging, tractional retinal detachment and vitral haemorrhage [238]. When a premature newborn is brought into a high oxygen environment, the growth of retinal blood vessels that expand from the optic nerve halts. After oxygen conditions are normalized, the inner retinal vasculature fails to regain the normal growth which in turn creates an avascular area and leads to neovascularisation, epiretinal angiogenesis and possibly loss of vision field [249]. Today, laser photocoagulation and intravitreal injections of bevacizumab or pegaptanib are used as therapeutic tools to manage ROP [25, 250, 251].

Interestingly, studies have shown that newborns that are suffering from ROP have had increased levels of serum prorenin [252], ocular renin [253, 254] as well as increased AT1R and AT2R expression [253]. Moreover, studies using animal models with oxygen induced retinopathy have shown that ACE inhibitors and AT1R antagonist treatments during the normal air conditions can reduce pathological angiogenesis in retina [253, 255-257]. However, the effects of treating retinal angiogenesis with AT2R antagonist and the possible role of AT2R in retinal vascular pathology are still uncertain [223, 254, 256, 258]. Nath *et al.* studied RAS involvement in ROP in infant patients (n=44) suffering from ROP as well as in rats with oxygen induced retinopathy. They found significantly higher levels of AGT, ACE1 and Ang II, in vitreous humor of infants suffering from ROP compared to healthy controls. Renin levels were also increased but the difference was found to be statistically not significant. In animal studies up-regulated mRNA expressions for renin, AGT, ACE1 and AT1R were found in retina of the rats suffering from oxygen induced retinopathy. Furthermore, ACE inhibitor (lisinopril) and ARB (telmisartan) were shown to suppress the over-activation induced vascular and retinal functional changes in ROP [25]. However, further research is needed to understand the involvement of RAS in the development and treatment of ROP.

## CONCLUSION

Interestingly, in addition to circulating RAS that is involved in BP regulation and inflammation, local tissue specific RAS has been identified in many organs of the human body including the eye. It has been detected in various parts of the eye even in the structures involved in aqueous humor formation and outflow. These intriguing observations should promote more research concentrating on the possible role of local RAS in aqueous humor dynamics and thus IOP regulation. However, factors such as retinal ganglion cell death due to other reasons than pressure might also be involved in glaucoma progression. Identification of the second counter-regulating axis of RAS (ACE2-Ang(1-7)-MasR) has already brought new opportunities into light, and compounds affecting Ang(1-7) synthesis as well as MasR and ACE2 activity offer new and exciting possibilities for ocular pharmacology in the future.

## Figures and Tables

**Table 1:** Renin-angiotensin system components in bulbar conjunctiva, cornea, trabecular meshwork, aqueous humor, iris, ciliary body and non-pigmented ciliary epithelium, of the human eye.

**RAS component**	**Bulbar conjunctiva**	**Cornea**	**Trabecular meshwork**	**Aqueous humor**	**Iris**	**Ciliary body/NPE**	**References**	
Prorenin	x	x		x	x	x	[24, 105-108]	
Renin	x	x			x	x	[105, 108]	
AGT	x	x		x	x	x	[105, 109, 110]	
ACE1	x	x	x	x	x	x	[104, 105, 110-116]	
ACE2				x			[104]	
Ang I				x	x	x	[117, 118]	
Ang II	x	x	x	x	x	x	[111, 117-119]	
Ang(1–7)			x	x		x	[93, 104]	
(P)RR	x	x			x	x	[105]	
AT, unknown subtype					x	x	[120, 121]	
AT1R	x	x			x	x	[105, 119, 122]	
AT2R					x	x	[119]	
AT4R								
MasR		x	x			x	[93]	

Table modified and updated from the table published by Holappa *et al.* [123]. ACE1, -2: Angiotensin converting enzyme 1, -2; AGT: Angiotensinogen; Ang I, -II: Angiotensin I, -II; Ang(1–7): Angiotensin (1–7); AT1R, -2R, -4R: Angiotensin II type 1, 2, 4 receptor; MasR: Mas receptor; NPE: Non-pigmented ciliary epithelium; (P)RR: (pro)renin receptor; RAS: Renin-angiotensin system.

**Table 2:** Renin-angiotensin system components in lens, tears and lacrimal gland, vitreous, optic nerve head and sclera of the human eye.

**RAS component**	**Lens**	**Tears/Lacrimal gland**	**Vitreous**	**Optic nerve head**	**Sclera**	**References**
Prorenin			x		x	[24, 105, 107]
Renin			x		x	[24, 105]
AGT			x		x	[105, 110]
ACE1		x	x	x	x	[105, 112, 114, 115, 124-127]
ACE2						
Ang I			x			[117]
Ang II			x	x		[111, 119]
Ang(1–7)						
(P)RR					x	[105]
AT, unknown subtype						
AT1R				x		[119]
AT2R				x		[119]
AT4R						
MasR						

Table modified and updated from the table published by Holappa *et al.* [123]. ACE1, -2: Angiotensin converting enzyme 1, -2; AGT: Angiotensinogen; Ang I, -II: Angiotensin I, -II; Ang(1–7): Angiotensin (1–7); AT1R, -2R, -4R: Angiotensin II type 1, 2, 4 receptor; MasR: Mas receptor; (P)RR: (pro)renin receptor; RAS: Renin-angiotensin system.
